# Survival benefit from immunocheckpoint inhibitors in stage IV non‐small cell lung cancer patients with brain metastases: A National Cancer Database propensity‐matched analysis

**DOI:** 10.1002/cam4.3675

**Published:** 2020-12-19

**Authors:** Shinkichi Takamori, Takefumi Komiya, Emily Powell

**Affiliations:** ^1^ Department of Thoracic Oncology National Hospital Organization Kyushu Cancer Center Fukuoka Japan; ^2^ Medical Oncology Parkview Cancer Institute Fort Wayne IN USA; ^3^ Parkview Research Center Mirro Center for Research and Innovation Fort Wayne IN USA; ^4^ Oncology Research Program Parkview Cancer Institute Fort Wayne IN USA

**Keywords:** immunology, medical oncology, metastasis, non‐small cell lung cancer

## Abstract

Immunocheckpoint inhibitors (ICIs) have become a standard pharmacological therapy in non‐small cell lung cancer (NSCLC). Because brain metastases (BMs) have historically been listed as exclusion criteria in previous clinical trials involving ICIs in advanced NSCLC, the survival benefit from ICI in NSCLC patients with BMs remains unclear. The National Cancer Database was queried for stage IV NSCLC patients with or without BMs between 2014 and 2015. Overall survival (OS) of stage IV NSCLC patients who received immunotherapy and that of stage IV NSCLC patients who did not receive immunotherapy were compared according to the presence or absence of BMs. Multivariable logistic analyses identified the clinical characteristics predictive of overall survival. A propensity score analysis was conducted with the aim of adjusting the potential biases arising from the clinical characteristics. This study included 42,512 patients with stage IV NSCLC; 11,810 patients with BMs and 30,702 patients without BMs. In univariate analysis, stage IV NSCLC patients with BMs treated with immunotherapy had a significantly longer OS than those without immunotherapy after propensity score matching (median OS: 12.8 vs 10.1 months, hazard ratio [HR]: 0.80, 95% confidence interval [CI]: 0.72–0.89, *p* < 0.0001). Multivariable Cox modeling after propensity score matching confirmed the survival benefit from ICI for stage IV NSCLC patients with BMs (HR: 0.75, 95% CI: 0.67–0.83, *p* < 0.0001). The HR in NSCLC patients without BMs treated with ICI compared with those without ICI was 0.77 (95% CI: 0.73–0.82, *p* < 0.0001). Survival in stage IV NSCLC patients with BMs was significantly improved by ICI treatment at levels comparable to those without BMs using a retrospective database. ICI may be one of the promising treatment options for stage IV NSCLC patients with BMs. These findings should be validated in future prospective studies.

## Graphical abstract

In previous clinical trials investigating efficacy of immune checkpoint inhibitors (ICIs) on advanced non‐small cell lung cancer (NSCLC), brain metastases (BMs) have been listed as exclusion criteria. Thus, the survival benefit from ICIs in NSCLC patients with BMs had been unclear. This retrospective study shows that overall survival in NSCLC patients with BMs was significantly improved by ICIs at levels comparable to those without BMs. ICIs may be promising treatment options for NSCLC patients with BMs.

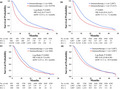

## 1 INTRODUCTION

Lung cancer is one of the most fatal malignancies worldwide, and non‐small cell lung cancer (NSCLC) accounts for 85% of lung cancer.^1^ Immunocheckpoint inhibitors (ICIs) targeting programmed cell death‐1 (PD‐1) or programmed cell death‐ligand 1 (PD‐L1) have been widely adopted in patients with NSCLC.^2^, ^3^, ^4^, ^5^, ^6^, ^7^, ^8^, ^9^, ^10^, ^11^ In patients with NSCLC, brain metastases (BMs) are diagnosed in approximately 20%–40% cases during the course of the disease.^12^, ^13^ However, in most previous clinical trials involving ICIs in advanced NSCLC, only patients with stable central nervous system metastases were eligible, and those with untreated symptomatic BMs were excluded.^2^, ^3^, ^4^, ^5^, ^6^, ^7^, ^8^, ^9^, ^10^, ^11^ The possible reasons for the exclusion criteria included use of corticosteroids and possibility of central nervous system pseudoprogression.^14^, ^15^

According to a previous clinical trial investigating the efficacy of nivolumab in nonsquamous advanced NSCLC (Checkmate 057), only 68 patients with BMs of total 582 patients (11.7%) were included.^2^ A subgroup analysis of the Checkmate 057 trial reported that the hazard ratio (HR) for overall survival (OS) in NSCLC patients with BMs treated with nivolumab compared with patients with BMs treated with docetaxel was 1.04 (95% confidence interval [CI], 0.62–1.76). However, a subgroup analysis of the OAK trial investigating 85 patients with BMs of total 850 patients (10.0%) showed that the HR for OS in NSCLC patients with BMs treated with atezolizumab in comparison to patients with BMs treated with docetaxel was 0.54 (95% CI, 0.31–0.94).^3^ Thus, the sample sizes of analyzed advanced NSCLC patients with BMs treated with ICIs in previous clinical trials were relatively small, and survival benefit from ICIs in such population remains unclear. Therefore, the aim of this study was to clarify the survival benefit from ICIs in advanced NSCLC patients with BMs using the National Cancer Database (NCDB).

## 2 MATERIALS AND METHODS

### 2.1 NCDB database

The NCDB is a joint project between the Commission on Cancer (CoC) of the American College of Surgeons and the American Cancer Society. The CoC’s NCDB and the hospitals participating in the CoC NCDB are the source of the deidentified data used herein; they have not verified and are not responsible for the statistical validity of the data analysis or the conclusions derived by the authors.

**Figure 1:**
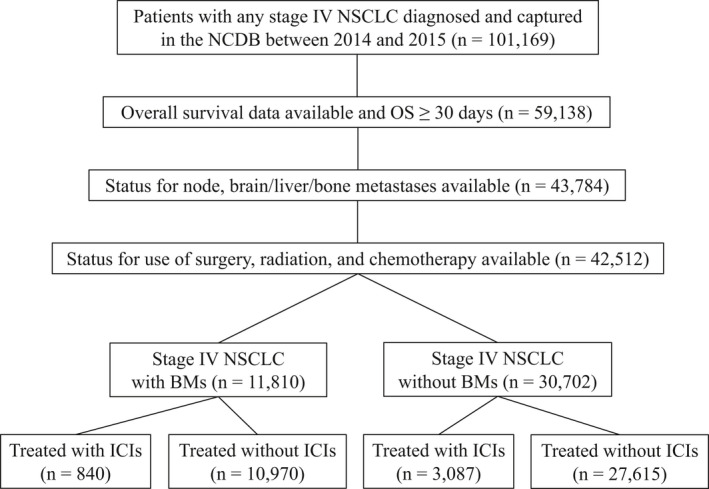
Study flow diagram of case eligibility

Patients with any stage IV NSCLC diagnosed and captured in the NCDB database between 2014 and 2015 were selected (n = 101,169). Of these, patients whose survival data were available and who survived at least 30 days past the date of diagnosis were included (n = 59,138). Patients with available status for node, brain/liver/bone metastases were then selected (n = 43,784). Of these, patients with available status for use of surgery, radiation, and chemotherapy were selected. Ultimately, 42,512 patients were eligible for final analysis with 11,810 stage IV NSCLC patients with BMs and 30,702 patients with stage IV NSCLC without BMs (Figure 1).

Clinical demographics such as age (<70 vs 70+), sex (male vs female), race (whites vs others), insurance (yes vs no), institutions (academic vs nonacademic), Charlson‐Deyo comorbidity score (0–1 vs 2–3), years of diagnosis (2014 vs 2015), histology (adenocarcinoma, not otherwise specified [NOS] vs others), nodal status (N0 vs N1+), bone metastasis (yes vs no), liver metastasis (yes vs no), surgery for primary lesion (yes vs no), radiation (yes vs no), and chemotherapy (yes vs no) were collected. Radiation includes all kinds and for both primary tumor and BMs. In stage IV NSCLC patients with BMs (n = 11,810), 9,688 patients received radiotherapy, including 7,289 for BMs and 2,399 for others. In stage IV NSCLC patients without BMs (n = 30,702), 10,717 patients received radiotherapy, including 265 for BMs and 10,452 for others.

### 2.2 Statistical analysis

The Kaplan‐Meier curves were compared using the log‐rank test. The associations between ICI (yes vs no) and clinical demographics were assessed by chi‐squared test. Univariate and multivariable Cox proportional hazards analyses were performed using JMP^®^ 14.0 (SAS Institute Inc., Cary, NC, USA). A propensity score analysis was conducted with the aim of reducing the bias of the retrospective nature of the study. In the analysis of patients with stage IV NSCLC with BMs, the propensity scores, which were calculated by a multivariable logistic analysis, included the following variables: age, sex, race, institution, Charlson‐Deyo score, histology, nodal status, bone metastasis, liver metastasis, surgery for primary lesion, radiation, and chemotherapy. The propensity score matching was performed using 1:1 nearest‐neighbor matching. In the analysis of those without BMs, the propensity scores included the following variables: age, sex, race, Charlson‐Deyo score, histology, nodal status, bone metastasis, liver metastasis, surgery for primary lesion, radiation, and chemotherapy. Propensity score matching analyses were performed according to XLSTAT software guideline. Finally, 840 matched patients with BMs from each group were included in the survival analysis. Similarly, 3,087 matched patients without BMs from each group were analyzed. In subgroup analyses, the HR for OS and its 95% CI after propensity score matching were provided. *p* < 0.05 was considered statistically significant.

## 3 RESULTS

### 3.1 Patient characteristics

A total of 42,512 cases were selected for the analysis. Clinical characteristics are shown in Supplemental Table S1. In total, 3,927 (9.2%) patients received ICIs. Use of ICIs was significantly more frequent in age <70, white race, academic institution, Charlson‐Deyo score 0–1, year of diagnosis in 2015, adenocarcinoma NOS histology, nodal status N1+, bone metastasis, no brain metastasis, no surgery for primary lesion, no radiation, and chemotherapy groups per univariate analysis (data not shown).

### 3.2 Associations between administration of ICIs and clinical factors in stage IV NSCLC patients with and without BMs

Table 1 and Supplemental Table S2 show the relationships between administration of ICIs and clinical factors with and without BMs, respectively. Before propensity score matching, in stage IV NSCLC patients with BMs, ICI use was significantly more frequent in age <70, Charlson‐Deyo score 0–1, year of diagnosis in 2015, adenocarcinoma NOS histology, nodal status N0, bone metastasis, no surgery for primary lesion, radiation, and chemotherapy groups per univariate analysis (Table 1). In stage IV NSCLC patients without BMs, administration of ICIs was significantly more frequent in age <70, nonacademic institution, Charlson‐Deyo score 0–1, year of diagnosis in 2015, adenocarcinoma NOS histology, nodal status N1+, bone metastasis, no surgery for primary lesion, no radiation, and chemotherapy groups per univariate analysis before propensity score matching (Supplemental Table S2). After propensity score matching, the distributions of the baseline patient characteristics between ICI‐administered patients and the other patients were well‐balanced.

**Table 1:** Clinical characteristics of stage IV non‐small cell lung cancer patients with BMs (n = 11,810)

Factors		Before propensity score matching (n = 11,810)			After propensity score matching (n = 1,680)		
		Immunotherapy, n (%)		*p* value	Immunotherapy, n (%)		*p* value
		Yes (n = 840)	No (n = 10,970)		Yes (n = 840)	No (n = 840)	
Age	<70	652 (78%)	7,336 (67%)	<0.0001	652 (78%)	657 (78%)	0.7687
	≥70	188 (22%)	3,634 (33%)		188 (22%)	183 (22%)	
Sex	male	422 (50%)	5,582 (51%)	0.7181	422 (50%)	418 (50%)	0.8453
	female	418 (50%)	5,388 (49%)		418 (50%)	422 (50%)	
Race	whites	718 (85%)	9,112 (83%)	0.0711	718 (85%)	718 (85%)	1.0000
	others	122 (15%)	1,858 (17%)		122 (15%)	122 (15%)	
Insurance status	uninsured	32 (4%)	387 (4%)	0.6705	32 (4%)	18 (2%)	0.0440
	insured	808 (96%)	10,583 (96%)		808 (96%)	822 (98%)	
Institution	academic	361 (43%)	4,370 (40%)	0.0734	361 (43%)	354 (42%)	0.7298
	others	479 (57%)	6,600 (60%)		479 (57%)	486 (58%)	
Charlson‐Deyo score	0–1	764 (91%)	9,674 (88%)	0.0159	764 (91%)	764 (91%)	0.6937
	≥2	76 (9%)	1,296 (12%)		76 (9%)	71 (9%)	
Year of diagnosis	2014	368 (44%)	5,563 (51%)	0.0001	368 (44%)	427 (51%)	0.0039
	2015	472 (56%)	5,407 (49%)		472 (56%)	413 (49%)	
Histology	adenocarcinoma NOS	638 (76%)	7,061 (64%)	<0.0001	638 (76%)	633 (75%)	0.7762
	others	202 (24%)	3,909 (36%)		202 (24%)	207 (25%)	
Nodal status	N0	671 (80%)	8,329 (76%)	0.0095	671 (80%)	675 (80%)	0.8068
	≥N1	169 (20%)	2,641 (24%)		169 (20%)	169 (20%)	
Bone metastasis	yes	350(42%)	3,456 (32%)	<0.0001	350(42%)	346 (41%)	0.8430
	no	490 (58%)	7,514 (68%)		490 (58%)	494 (59%)	
Liver metastasis	yes	124 (15%)	1,779 (16%)	0.2689	124 (15%)	115 (14%)	0.5296
	no	716(85%)	9,191 (84%)		716(85%)	725 (86%)	
Surgery for primary lesion	yes	15 (2%)	336 (3%)	0.0357	15 (2%)	15 (2%)	1.0000
	no	825 (98%)	10,634 (97%)		825 (98%)	825 (98%)	
Radiation	yes	746 (89%)	8,942 (82%)	<0.0001	746 (89%)	750 (89%)	0.7547
	no	94 (11%)	2,028 (18%)		94 (11%)	90 (11%)	
Chemotherapy	yes	760 (90%)	6,618 (60%)	<0.0001	760 (90%)	760 (90%)	1.0000
	no	80 (10%)	4,352 (40%)		80 (10%)	80 (10%)	

Abbreviations: BM, brain metastasis; NOS, not otherwise specified.

### 3.3 Univariate survival analyses in stage IV NSCLC patients according to BMs

**Figure 2:**
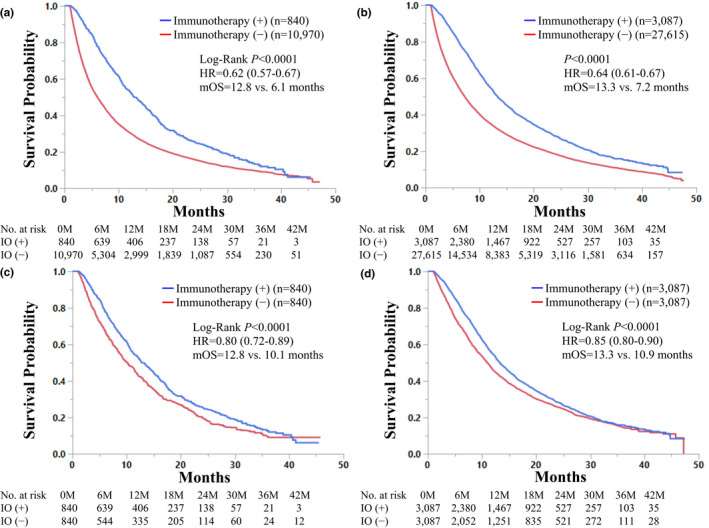
Kaplan‐Meier curves comparing overall survival in stage IV non‐small cell lung cancer (NSCLC) patients treated with immunocheckpoint inhibitors (ICIs) and that of stage IV NSCLC patients who did not receive ICIs according to brain metastasis status are shown. (A) In patients with brain metastases, ICI‐administered patients had a significantly longer overall survival than the other patients. (B) In patients without brain metastases, ICI‐administered patients had a significantly longer overall survival than the other patients. (C) After propensity score matching, in patients with brain metastases, ICI‐administered patients had a significantly longer overall survival than the other patients. (D) In patients without brain metastases, ICI‐administered patients had a significantly longer overall survival than the other patients after propensity score matching

The Kaplan‐Meier curve comparing OS according to ICI status in patients with stage IV NSCLC (total cohort) is shown in Supplemental Figure S1. Patients who received ICIs had a significantly longer OS than those who did not (median OS: 13.3 vs 6.9 months, HR for death: 0.63 [95% CI: 0.60–0.65], *p* < 0.0001). The Kaplan‐Meier curves of OS in stage IV NSCLC patients treated with ICIs according to BMs status are shown in Figure 2. In patients with BMs, ICI‐administered patients had a significantly longer OS than the other patients (median OS: 12.8 vs 6.1 months, HR for death: 0.62 [95% CI: 0.57–0.67], *p* < 0.0001, Figure 2A). Similarly, in patients without BMs, ICI‐administered patients had a significantly longer OS than the other patients (Figure 2B). As shown in Figure 2C,D, these findings remained significant after propensity score matching. Stage IV NSCLC patients with BMs treated with ICIs had a significantly longer OS than those without ICIs after propensity score matching (median OS: 12.8 vs 10.1 months, HR for death: 0.80 [95% CI: 0.72–0.89], *p* < 0.0001, Figure 2C). The HR for death in stage IV NSCLC patients without BMs treated with ICIs compared with those without ICIs was 0.85 (95% CI, 0.80–0.90, Figure 2D).

### 3.4 Univariate and multivariable analyses of OS in stage IV NSCLC patients according to BMs

The results of univariate and multivariable analyses for OS in stage IV NSCLC patients with and without BMs are shown in Table 2 and Supplemental Table S2, respectively. Before propensity score matching, ICI was an independent factor for predicting longer OS (HR for death: 0.70 [95% CI: 0.65–0.77], *p* < 0.0001, Ta). After propensity score matching, multivariable analysis of OS in stage IV NSCLC patients with BMs demonstrated that younger age, female, races other than white, academic institution, Charlson‐Deyo score 0–1, adenocarcinoma NOS histology, no bone metastasis, no liver metastasis, chemotherapy, and ICI were independent predictors for longer OS (HR for death with ICI: 0.75 [95% CI: 0.67–0.83], *p* < 0.0001, Table 2). In patients with stage IV NSCLC patients without BMs, multivariable analysis of OS before propensity score matching revealed that ICI was an independent factor for predicting longer OS (HR for death: 0.73 [95% CI: 0.70–0.76], *p* < 0.0001, Supplemental Table S2). Multivariable analysis of OS after propensity score matching showed that younger age, female, races other than white, academic institution, Charlson‐Deyo score 0–1, adenocarcinoma NOS histology, nodal status N0, no bone metastasis, no liver metastasis, surgery for primary lesion, no radiation therapy, chemotherapy, and ICI were independent predictive factors for longer OS (HR for death with ICI: 0.77 [95% CI: 0.73–0.82], *p* < 0.0001, Supplemental Table S2).

**Table 2:** Clinical characteristics of stage IV non‐small cell lung cancer patients without BMs before and after propensity score matching (n = 30,702)

Factors		Before propensity score matching (n = 30,702)			After propensity score matching (n = 6,174)		
		Immunotherapy, n (%)		*p* value	Immunotherapy, n (%)		*p* value
		Yes (n = 3,087)	No (n = 27,615)		Yes (n = 3,087)	No (n = 3,087)	
Age	<70	1,957 (63%)	13,522 (49%)	<0.0001	1,957 (63%)	1,959 (63%)	0.9579
	≥70	1,130 (37%)	14,093 (51%)		1,130 (37%)	1,128 (37%)	
Sex	male	1,629 (53%)	15,008 (54%)	0.0950	1,629 (53%)	1,628 (53%)	0.9800
	female	1,458 (47%)	12,607 (46%)		1,458 (47%)	1,459 (47%)	
Race	whites	2,605 (84%)	22,926 (83%)	0.0544	2,605 (84%)	2,601 (84%)	0.8887
	others	482 (16%)	4689 (17%)		482 (16%)	486 (16%)	
Insurance status	uninsured	67 (2%)	688 (2%)	0.2748	67 (2%)	48 (2%)	0.0737
	insured	3,020 (98%)	26,927 (98%)		3,020 (98%)	3,039 (98%)	
Institution	academic	1,122 (36%)	9,141 (33%)	0.0003	1,122 (36%)	1,513 (49%)	<0.0001
	others	1,965 (64%)	18,474 (67%)		1,965 (64%)	1,574 (51%)	
Charlson‐Deyo score	0–1	2,785 (90%)	23,571 (85%)	<0.0001	2,785 (90%)	2,791 (90%)	0.7963
	≥2	302 (10%)	4,044 (15%)		302 (10%)	296 (10%)	
Year of diagnosis	2014	1,287 (42%)	14,123 (51%)	<0.0001	1,287 (42%)	1,600 (52%)	<0.0001
	2015	1,800 (58%)	13,492 (49%)		1,800 (58%)	1,487 (48%)	
Histology	adenocarcinoma NOS	2,325 (75%)	14,980 (54%)	<0.0001	2,325 (75%)	2,323 (75%)	0.9530
	others	762 (25%)	12,635 (46%)		762 (25%)	764 (25%)	
Nodal status	N0	722 (23%)	7,665 (28%)	<0.0001	722 (23%)	717 (23%)	0.8804
	≥N1	2,365 (77%)	19,950 (72%)		2,365 (77%)	2,370 (77%)	
Bone metastasis	yes	1,455 (47%)	10,639 (39%)	<0.0001	1,455 (47%)	1,453 (47%)	0.9593
	no	1,632 (53%)	16,976 (61%)		1,632 (53%)	1,634 (53%)	
Liver metastasis	yes	493 (16%)	4,418 (16%)	0.9674	493 (16%)	485 (16%)	0.7804
	no	2,594 (84%)	23,197 (84%)		2,594 (84%)	2,602 (84%)	
Surgery for primary lesion	yes	83 (3%)	954 (3%)	0.0255	83 (3%)	86 (3%)	0.8150
	no	3,004 (97%)	26,661 (97%)		3,004 (97%)	3,001 (97%)	
Radiation	yes	932 (30%)	9,785 (35%)	<0.0001	932 (30%)	932 (30%)	1.0000
	no	2,155 (70%)	17,830 (65%)		2,155 (70%)	2,155 (70%)	
Chemotherapy	yes	2,834 (91%)	16,907 (61%)	<0.0001	2,834 (91%)	2,833 (92%)	0.9630
	no	253 (9%)	10,708 (39%)		253 (9%)	254 (8%)	

Abbreviations: BM, brain metastasis; NOS, not otherwise specified.

### 3.5 Subgroup analyses for OS in stage IV NSCLC patients according to clinical factors

**Figure 3:**
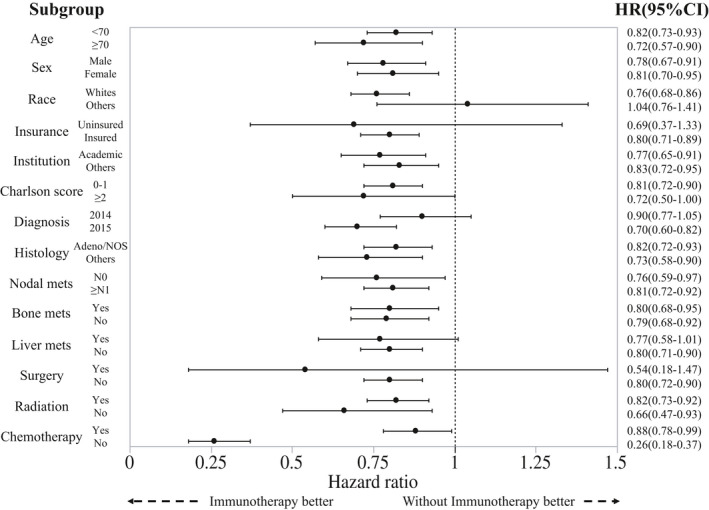
Subgroup analyses of overall survival in stage IV non‐small cell lung cancer patients with brain metastases according to each clinical factor are shown. HR, hazard ratio; NOS,, adenocarcinoma, not otherwise specified

The results of subgroup analyses for OS in stage IV NSCLC patients with and without BMs after propensity score matching according to each clinical factor are shown in Figure 3 and Supplemental Figure S2. As shown in Figure 3, in stage IV NSCLC patients with BMs, administration of ICIs was associated with longer OS in each subgroup analysis except for races other than white. ICI treatment was also related to better prognosis in each group in stage IV NSCLC patients without BMs (Supplemental Figure S2).

## 4 DISCUSSION

In patients with many types of cancer, BMs are often difficult concerns to be resolved due to the inadequate delivery of antitumor agents through blood‐brain barrier, difficulty in access to the BMs, and neurological symptoms resulting in a decreased performance status.^16^ Regarding ICIs, given that the brain is an immune‐privileged sanctuary,^16^ whether ICIs can provoke the anti‐PD‐1/PD‐L1 reactions at the tumor microenvironment in the brain remained unknown, even if the ICIs could penetrate the blood‐brain barrier.^17^, ^18^, ^19^ Additionally, in clinical trial settings, NSCLC patients with BMs were often underrepresented in trials because a) most patients with BMs are excluded from trial due to specifications in the exclusion criteria,^20^ and b) physician investigators were reluctant to enroll them due to safety concerns.^21^ Thus, the efficacy of ICIs in NSCLC patients with BMs had not been well elucidated. However, several recent studies have implied that T cells and tumor‐infiltrating lymphocytes can be delivered to the brain, and were associated with improved survival and better responses to ICIs.^22^, ^23^, ^24^ In a clinical setting, a previous retrospective study investigating 409 NSCLC patients with BMs treated with nivolumab showed that the intracranial objective response rate and disease control rate were 17% and 39%, respectively.^25^ In addition, the previous phase II trial investigating efficacy of pembrolizumab for PD‐L1‐positive NSCLC patients with BMs showed that the intracranial objective response rate was 29.7%.^26^ Our results that ICIs contributed to significant survival benefit in stage IV NSCLC patients with BMs were in line with the findings in these recent studies.

In this retrospective study with propensity score matching, we demonstrated that stage IV NSCLC patients treated with ICIs had a significantly longer OS than those without ICIs independent of the presence or absence of BMs. In this large cohort of stage IV NSCLC patients, 840 patients had BMs at the beginning of treatment with ICIs. Of note, the sample size of NSCLC patients with BMs receiving ICIs was larger than that reported in the previous representative phase III clinical trials (9–73 patients).^2^, ^3^, ^4^, ^5^, ^6^, ^7^, ^8^, ^9^, ^10^, ^11^ The representative Phase III clinical trials investigating NSCLC patients treated with ICIs are summarized in Supplemental Table S3. NSCLC patients with BMs were included in only 5.5–17.5% of the total cohort.^2^, ^3^, ^4^, ^5^, ^6^, ^7^, ^8^, ^9^, ^10^ The HRs in NSCLC patients with BMs treated with ICIs compared with those without ICIs were available in four previous clinical trials (Checkmate 057, KEYNOTE‐024, KEYNOTE‐189, and OAK), and they were ranging from 0.36 to 1.04.^2^, ^3^, ^4^, ^27^ Thus, survival benefit from ICIs in NSCLC patients with BMs was analyzed with the small number of patients in these previous trials, and that these results were controversial. In this study, the multivariable survival analysis showed that the HR in NSCLC patients with BMs treated with ICIs compared with those without ICIs was 0.75 (95% CI: 0.67–0.83) after propensity score matching (Table 2). It is notable that the HR in NSCLC patients without BMs treated with ICIs in comparison to those without ICIs was 0.77 (95% CI: 0.73–0.82). These data indicate that NSCLC patients with BMs may be good candidates for ICIs. Our data also suggested that ICIs improved OS in NSCLC patients with BMs by approximately 2.7 months (Figure 2C). Given that the median OS of stage IV NSCLC patients with BMs who were not treated by ICIs was 10.1 months, the survival benefit from ICIs would be clinically meaningful in this population. Thus, ICIs are one of the promising therapeutic strategies in stage IV NSCLC patients with BMs (Table 3).

**Table 3:** Multivariable analyses of overall survival in stage IV non‐small cell lung cancer patients with BMs before and after propensity score matching

Factors		Before propensity score matching (n = 11,810)		After propensity score matching (n = 1,680)	
		Univariate	Multivariable	Univariate	Multivariable
		HR (95% CI)	HR (95% CI)	HR (95% CI)	HR (95% CI)
		*p* value	*p* value	*p* value	*p* value
Age	<70	0.70 (0.67–0.73)	0.83 (0.80–0.87)	0.79 (0.69–0.90)	0.84 (0.74–0.96)
	≥70	<0.0001	<0.0001	0.0005	0.0106
Sex	female	0.84 (0.81–0.87)	0.86 (0.83–0.89)	0.86 (0.77–0.96)	0.86 (0.77–0.96)
	male	<0.0001	<0.0001	0.0074	0.0085
Race	others	0.85 (0.80–0.90)	0.85 (0.81–0.90)	0.72 (0.61–0.85)	0.77 (0.65–0.91)
	whites	<0.0001	<0.0001	<0.0001	0.0021
Insurance status	insured	1.02 (0.92–1.14)	1.00 (0.90–1.12)	0.87 (0.65–1.20)	0.90 (0.67–1.25)
	uninsured	0.7003	0.9735	0.3704	0.5277
Institution	academic	0.80 (0.77–0.83)	0.82 (0.79–0.85)	0.82 (0.73–0.91)	0.80 (0.71–0.90)
	others	<0.0001	<0.0001	0.0003	0.0001
Charlson‐Deyo score	0–1	0.73 (0.69–0.78)	0.83 (0.78–0.88)	0.71 (0.59–0.86)	0.76 (0.63–0.93)
	≥2	<0.0001	<0.0001	0.0006	0.0067
Year of diagnosis	2015	0.95 (0.91–0.99)	0.97 (0.93–1.01)	0.97 (0.87–1.08)	0.98 (0.88–1.10)
	2014	0.0074	0.1533	0.5978	0.7533
Histology	adenocarcinoma NOS	0.78 (0.75–0.81)	0.81 (0.77–0.84)	0.79 (0.70–0.90)	0.82 (0.72–0.93)
	others	<0.0001	<0.0001	0.0004	0.0029
Nodal status	N0	0.88 (0.84–0.92)	0.78 (0.75–0.82)	0.92 (0.80–1.05)	0.92 (0.80–1.06)
	≥N1	<0.0001	<0.0001	0.2192	0.2503
Bone metastasis	no	0.81 (0.78–0.85)	0.79 (0.76–0.83)	0.74 (0.66–0.82)	0.76 (0.68–0.86)
	yes	<0.0001	<0.0001	<0.0001	<0.0001
Liver metastasis	no	0.68 (0.66–0.70)	0.72 (0.69–0.77)	0.65 (0.56–0.76)	0.71 (0.61–0.83)
	yes	<0.0001	<0.0001	<0.0001	<0.0001
Surgery for primary lesion	yes	0.47 (0.41–0.53)	0.51 (0.45–0.59)	0.62 (0.37–0.96)	0.64 (0.39–1.04)
	no	<0.0001	<0.0001	0.0322	0.0744
Radiation	yes	0.75 (0.71–0.79)	0.98 (0.93–1.03)	0.97 (0.81–1.17)	1.01 (0.84–1.21)
	no	<0.0001	0.3724	0.7466	0.9217
					
Chemotherapy	yes	0.38 (0.36–0.39)	0.38 (0.36–0.39)	0.61 (0.51–0.73)	0.58 (0.48–0.70)
	no	<0.0001	<0.0001	<0.0001	<0.0001
Immunotherapy	yes	0.62 (0.57–0.67)	0.70 (0.65–0.77)	0.80 (0.72–0.89)	0.75 (0.67–0.83)
	no	<0.0001	<0.0001	<0.0001	<0.0001

Abbreviations: BM, brain metastasis; NOS, not otherwise specified.

With regard to the subgroup survival analyses, ICI was associated with longer OS in each subgroup analysis except for races other than white in stage IV NSCLC patients with BMs (Figure 2). A previous study investigating the POPLAR and OAK trials elucidated that progression‐free survival was shorter in Asian patients compared to white race patients (12‐months survival rate: 12.9% vs 20.9%), which was similar to our findings.^28^ In that study, *epidermal growth factor receptor* (*EGFR*) mutation was investigated, and a different profile in relation to *EGFR* mutant rates was observed between Asian and white race patients (23.8% vs 8.5%).^28^ The *EGFR* mutational status has been reported to be associated with negative treatment outcomes in NSCLC patients treated with anti‐PD‐1 therapy.^29^, ^30^ The difference in *EGFR* status may at least partly explain the reason why other races did not benefit from ICIs in comparison to white race. Excluding the races, ICI was associated with longer OS in each subgroup analysis in stage IV NSCLC patients with BMs. Regarding the survival benefit from radiotherapy in stage IV NSCLC patients with BMs receiving ICI, the subgroup analysis of OS according to radiation in stage IV NSCLC patients with BMs showed that the HR in patients who had received radiotherapy and treated with ICI compared with those without ICI was 0.82 (95% CI: 0.73–0.92). Given that the HR in stage IV NSCLC patients who had not received radiotherapy and treated with ICI compared with those without ICI was 0.66 (95% CI: 0.47–0.93), the interaction between radiation and ICI was not suggested in this study.

There are some limitations in association with our study. First, NCDB lack several prognostic factors including patients’ performance status, use of corticosteroids, and second/third line treatments. These potential confounding factors may affect the patients’ survival. However, to the best of our knowledge, this study reports the largest collection of NSCLC patients with BMs treated with ICIs. Second, in this study, how to identify the stage IV NSCLC patients who will benefit from ICI treatment remains unknown. Previous studies have suggested several potential predictive biomarkers for efficacy of ICIs on BMs from NSCLC.^22^, ^31^, ^32^ Further studies investigating predictive factors for the response to ICI treatment may clarify the NSCLC patients with BMs who will benefit from such treatment.

In conclusion, this study suggests that survival in stage IV NSCLC patients with BMs was significantly improved by ICI treatment at levels comparable to those without BMs using a retrospective database. ICI may be one of the promising treatment options for stage IV NSCLC patients with BMs. These findings should be validated in future prospective studies.

## CONFLICTS OF INTEREST

Takefumi Komiya received travel fee from Merck. Shinkichi Takamori and Emily Powell declare no conflicts of interest in association with this study.

## AUTHOR CONTRIBUTIONS

Shinkichi Takamori wrote the manuscript. Takefumi Komiya significantly contributed to all of the ideas of this study and methods of analyzing the data. Emily Powell supervised the writing of the manuscript.

## Supporting information

Fig S1

Fig S2

Table S1

Table S2

Table S3

## Data Availability

The data that support the findings of this study are available from the corresponding author upon reasonable request.
