# Supplementary figures and images for: Survival benefit from immunocheckpoint inhibitors in stage IV non‐small cell lung cancer patients with brain metastases: A National Cancer Database propensity‐matched analysis
Source: Cancer Med. 2020 Dec 19;10(3):923–32. doi: 10.1002/cam4.3675 (PMC7897968; doi:10.1002/cam4.3675)

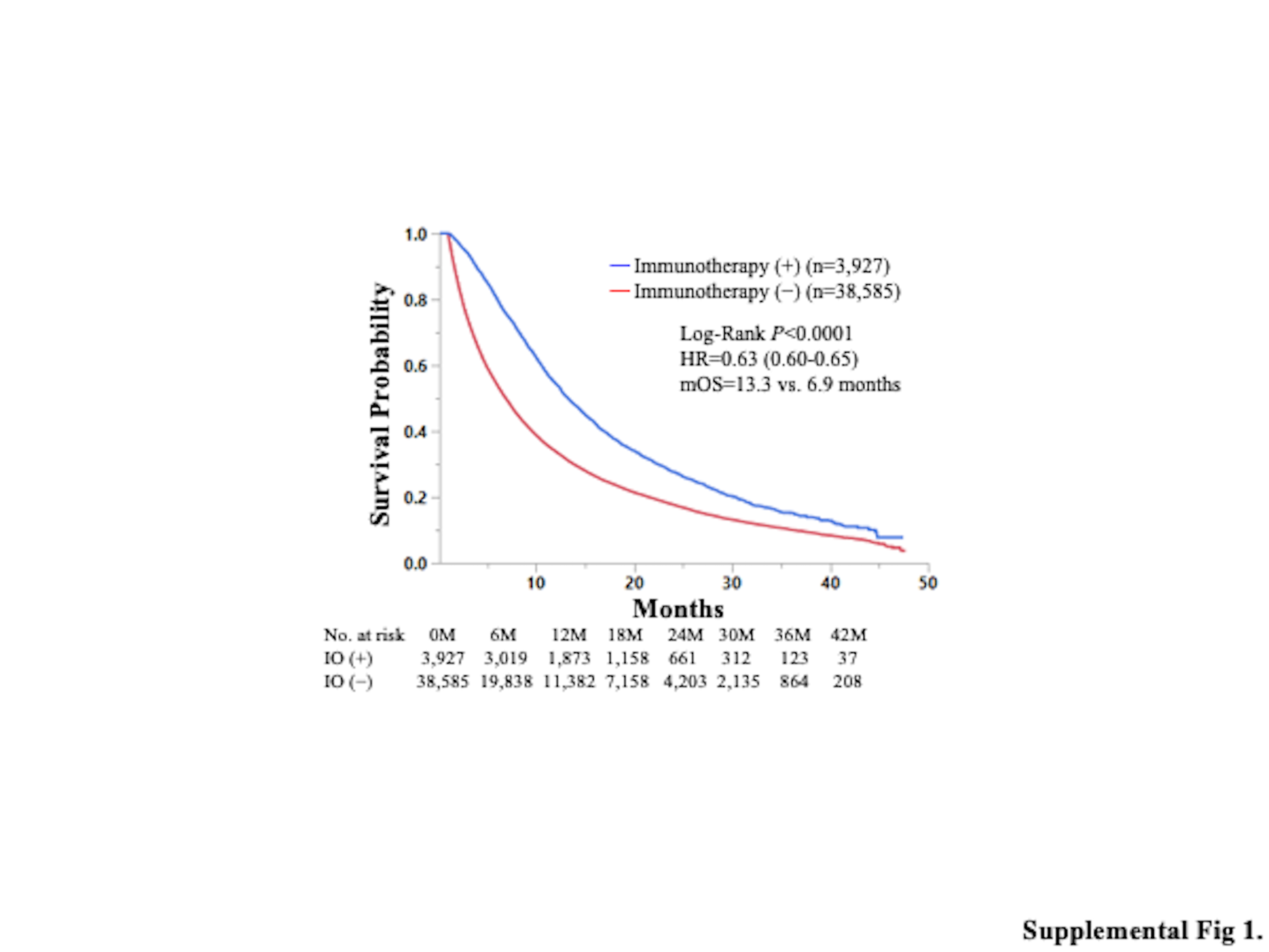

Supplement: Supplementary file 1 — Fig S1 [file CAM4-10-923-s001.tiff]

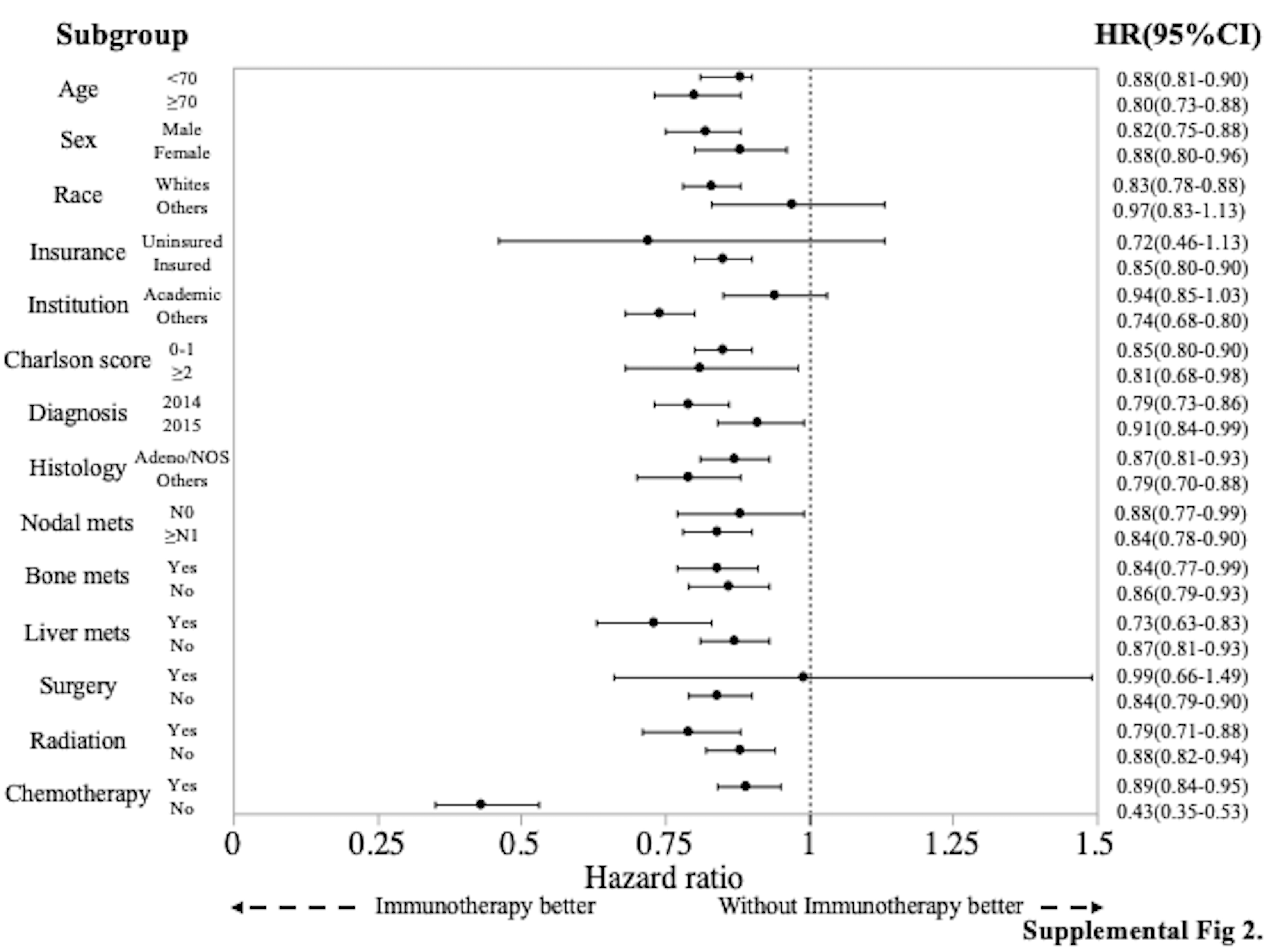

Supplement: Supplementary file 2 — Fig S2 [file CAM4-10-923-s002.tiff]
