# Supplementary material for: Survival benefit from immunocheckpoint inhibitors in stage IV non‐small cell lung cancer patients with brain metastases: A National Cancer Database propensity‐matched analysis
Source: Cancer Med. 2020 Dec 19;10(3):923–32. doi: 10.1002/cam4.3675 (PMC7897968; doi:10.1002/cam4.3675)
Supplement: Supplementary file 3 — Table S1 [file CAM4-10-923-s003.docx]

**Supplemental Table 1.** Clinical characteristics of stage IV NSCLC patients with or without BMs (n = 42,512).

| **Factors** |  | **Value or no. of patients** | ***P* value** |
| --- | --- | --- | --- |
| Age | <70 | 23,467 (55%) | <0.0001 |
|  | ≥70 | 19,045 (45%) |  |
|  |  |  |  |
| Sex | male | 22,641 (53%) | 0.1746 |
|  | female | 19,871 (47%) |  |
|  |  |  |  |
| Race | whites | 35,361 (83%) | 0.0113 |
|  | others | 7,151 (17%) |  |
|  |  |  |  |
| Insurance status | uninsured | 1,174 (3%) | 0.3342 |
|  | insured | 41,338 (97%) |  |
|  |  |  |  |
| Institution | academic | 14,994 (35%) | 0.0006 |
|  | others | 27,518 (65%) |  |
|  |  |  |  |
| Charlson-Deyo score | 0-1 | 36,794 (87%) | <0.0001 |
|  | ≥2 | 57,18 (13%) |  |
|  |  |  |  |
| Year of diagnosis | 2014 | 21,341 (50%) | <0.0001 |
|  | 2015 | 21,171 (50%) |  |
|  |  |  |  |
| Histology | adenocarcinoma NOS | 25,004 (59%) | <0.0001 |
|  | others | 17,508 (41%) |  |
|  |  |  |  |
| Nodal status | N0 | 11,197 (26%) | <0.0001 |
|  | ≥N1 | 31,315 (74%) |  |
|  |  |  |  |
| Bone metastasis | yes | 15,900 (37%) | <0.0001 |
|  | no | 26,612 (63%) |  |
|  |  |  |  |
| Liver metastasis | yes | 6,814 (16%) | 0.5702 |
|  | no | 35,698 (84%) |  |
|  |  |  |  |
| Surgery for primary lesion | yes | 11,810 (28%) | <0.0001 |
|  | no | 30,702 (72%) |  |
|  |  |  |  |
| Radiation | yes | 1,388 (3%) | 0.0044 |
|  | no | 41,124 (97%) |  |
|  |  |  |  |
| Chemotherapy | yes | 20,405 (48%) | <0.0001 |
|  | no | 22,107 (52%) |  |
|  |  |  |  |
| Immunotherapy | yes | 3,927 (9%) | <0.0001 |
|  | no | 38,585 (91%) |  |

BM, brain metastasis; NOS, not otherwise specified.
