# Supplementary material for: Survival benefit from immunocheckpoint inhibitors in stage IV non‐small cell lung cancer patients with brain metastases: A National Cancer Database propensity‐matched analysis
Source: Cancer Med. 2020 Dec 19;10(3):923–32. doi: 10.1002/cam4.3675 (PMC7897968; doi:10.1002/cam4.3675)
Supplement: Supplementary file 4 — Table S2 [file CAM4-10-923-s004.docx]

**Supplemental Table 2**. Multivariable analyses of overall survival in stage IV non-small cell lung cancer patients without BMs before and after propensity score matching.

| **Factors** | | **Before propensity score matching (n = 30,702)** | | |  | **After propensity score matching (n = 6,174)** | | |
| --- | --- | --- | --- | --- | --- | --- | --- | --- |
|  |  | **Univariate** |  | **Multivariable** |  | **Univariate** |  | **Multivariable** |
|  |  | **HR (95% CI)** |  | **HR (95% CI)** |  | **HR (95% CI)** |  | **HR (95% CI)** |
|  |  | ***P* value** |  | ***P* value** |  | ***P* value** |  | ***P* value** |
| Age | <70 | 0.80 (0.78-0.82) |  | 0.91 (0.89-0.94) |  | 0.86 (0.81-0.91) |  | 0.88 (0.83-0.93) |
|  | ≥70 | <0.0001 |  | <0.0001 |  | <0.0001 |  | <0.0001 |
|  |  |  | | |  |  | | |
| Sex | female | 0.81 (0.79-0.83) |  | 0.83 (0.81-0.85) |  | 0.77 (0.72-0.81) |  | 0.79 (0.74-0.84) |
|  | male | <0.0001 |  | <0.0001 |  | <0.0001 |  | <0.0001 |
|  |  |  | | |  |  | | |
| Race | others | 0.87 (0.84-0.90) |  | 0.89 (0.86-0.92) |  | 0.79 (0.72-0.86) |  | 0.84 (0.77-0.91) |
|  | whites | <0.0001 |  | <0.0001 |  | <0.0001 |  | <0.0001 |
|  |  |  | | |  |  | | |
| Insurance status | insured | 0.92 (0.84-1.00) |  | 0.88 (0.81-0.96) |  | 0.95 (0.77-1.20) |  | 0.95 (0.76-1.19) |
|  | uninsured | 0.0392 |  | 0.0038 |  | 0.6649 |  | 0.6367 |
|  |  |  | | |  |  | | |
| Institution | academic | 0.82 (0.79-0.84) |  | 0.85 (0.83-0.87) |  | 0.78 (0.73-0.82) |  | 0.75 (0.71-0.80) |
|  | others | <0.0001 |  | <0.0001 |  | <0.0001 |  | <0.0001 |
|  |  |  | | |  |  | | |
| Charlson-Deyo score | 0-1 | 0.73 (0.71-0.76) |  | 0.80 (0.77-0.82) |  | 0.76 (0.69-0.84) |  | 0.79 (0.71-0.87) |
|  | ≥2 | <0.0001 |  | <0.0001 |  | <0.0001 |  | <0.0001 |
|  |  |  | | |  |  | | |
| Year of diagnosis | 2015 | 0.98 (0.96-1.01) |  | 0.98 (0.96-1.00) |  | 1.00 (0.94-1.06) |  | 0.99 (0.93-1.05) |
|  | 2014 | 0.1616 |  | 0.1516 |  | 0.9040 |  | 0.7527 |
|  |  |  | | |  |  | | |
| Histology | adenocarcinoma NOS | 0.87 (0.84-0.89) |  | 0.95 (0.92-0.97) |  | 0.84 (0.79-0.90) |  | 0.90 (0.84-0.97) |
|  | others | <0.0001 |  | <0.0001 |  | <0.0001 |  | 0.0031 |
|  |  |  | | |  |  | | |
| Nodal status | N0 | 0.80 (0.77-0.82) |  | 0.74 (0.71-0.76) |  | 0.77 (0.71-0.82) |  | 0.77 (0.71-0.82) |
|  | ≥N1 | <0.0001 |  | <0.0001 |  | <0.0001 |  | <0.0001 |
|  |  |  | | |  |  | | |
| Bone metastasis | no | 0.73 (0.71-0.74) |  | 0.73 (0.71-0.75) |  | 0.70 (0.66-0.74) |  | 0.75 (0.70-0.80) |
|  | yes | <0.0001 |  | <0.0001 |  | <0.0001 |  | <0.0001 |
|  |  |  | | |  |  | | |
| Liver metastasis | no | 0.68 (0.66-0.70) |  | 0.72 (0.70-0.74) |  | 0.62 (0.57-0.66) |  | 0.66 (0.61-0.71) |
|  | yes | <0.0001 |  | <0.0001 |  | <0.0001 |  | <0.0001 |
|  |  |  | | |  |  | | |
| Surgery for primary lesion | yes | 0.48 (0.44-0.52) |  | 0.51 (0.47-0.56) |  | 0.59 (0.48-0.72) |  | 0.74 (0.60-0.91) |
|  | no | <0.0001 |  | <0.0001 |  | <0.0001 |  | 0.0043 |
|  |  |  | | |  |  | | |
| Radiation | yes | 1.11 (1.08-1.14) |  | 1.01 (0.99-1.04) |  | 1.23 (1.15-1.31) |  | 1.07 (1.00-1.15) |
|  | no | <0.0001 |  | 0.3422 |  | <0.0001 |  | 0.0429 |
|  |  |  | | |  |  | | |
| Chemotherapy | yes | 0.45 (0.44-0.47) |  | 0.44 (0.43-0.45) |  | 0.63 (0.57-0.70) |  | 0.57 (0.52-0.64) |
|  | no | <0.0001 |  | <0.0001 |  | <0.0001 |  | <0.0001 |
|  |  |  | | |  |  | | |
| Immunotherapy | yes | 0.64 (0.61-0.67) |  | 0.73 (0.70-0.76) |  | 0.85 (0.80-0.90) |  | 0.77 (0.73-0.82) |
|  | no | <0.0001 |  | <0.0001 |  | <0.0001 |  | <0.0001 |

BM, brain metastasis; NOS, not otherwise specified.
