# Supplementary material for: Survival benefit from immunocheckpoint inhibitors in stage IV non‐small cell lung cancer patients with brain metastases: A National Cancer Database propensity‐matched analysis
Source: Cancer Med. 2020 Dec 19;10(3):923–32. doi: 10.1002/cam4.3675 (PMC7897968; doi:10.1002/cam4.3675)
Supplement: Supplementary file 5 — Table S3 [file CAM4-10-923-s005.docx]

**Supplemental Table 3.** Summary of representative Phase II/III clinical trials investigating non-small cell lung cancer patients treated by immune checkpoint inhibitors.

| **Clinical trial** | **Histology** | **Number of patients with BMs/total cohort** | **PD-L1** | **Experimental arm** | **Control arm** | **Hazard ratio**  **(95% CI)** |
| --- | --- | --- | --- | --- | --- | --- |
| Checkmate 017  (NCT01642004) | Sq | 17/272 (6.3%) | All comer | Nivolumab | Docetaxel | NA |
|  |  |  |  |  |  |  |
| Checkmate 026  (NCT02041533) | NSCLC | 69/541 (12.8%) | ≥5% | Nivolumab | Platinum-based drug | NA |
|  |  |  |  |  |  |  |
| Checkmate 057  (NCT01673867) | Non-Sq NSCLC | 68/582 (11.7%) | All comer | Nivolumab | Docetaxel | 1.04  (0.62-1.76) |
|  |  |  |  |  |  |  |
| KEYNOTE-010  (NCT01905657) | NSCLC | NA | ≥1% | Pembrolizumab | Docetaxel | NA |
|  |  |  |  |  |  |  |
| KEYNOTE-024  (NCT02142738) | NSCLC | 28/305 (9.2%) | ≥50% | Pembrolizumab | Platinum-based drug | 0.73  (0.20-2.62) |
|  |  |  |  |  |  |  |
| KEYNOTE-042  (NCT02220894) | NSCLC | 70/1,274 (5.5%) | ≥1% | Pembrolizumab | Platinum-based drug | NA |
|  |  |  |  |  |  |  |
| KEYNOTE-189  (NCT02578680) | Non-Sq NSCLC | 108/616 (17.5%) | All comer | Pembrolizumab plus platinum-based drug and PEM | Platinum-based drug and PEM | 0.36  (0.20-0.62) |
|  |  |  |  |  |  |  |
| KEYNOTE-407  (NCT02775435) | Sq | 44/559 (7.9%) | All comer | Pembrolizumab plus CBDCA and either PTX or nab-PTX | CBDCA and either PTX or nab-PTX | NA |
|  |  |  |  |  |  |  |
| OAK  (NCT02008227) | NSCLC | 85/850 (10.0%) | All comer | Atezolizumab | Docetaxel | 0.54  (0.31-0.94) |
|  |  |  |  |  |  |  |
| Impower 130  (NCT02367781) | Non-Sq NSCLC | NA | All comer | Atezolizumab plus carboplatin and either PTX or nab-PTX | Carboplatin and either PTX or nab-PTX | NA |
|  |  |  |  |  |  |  |
| Impower 150  (NCT02366143) | Non-Sq NSCLC | NA | All comer | Atezolizumab plus CBDCA, PTX, and BEV | CBDCA, PTX, and BEV | NA |

BM, brain metastasis; PD-L1, programmed cell death-ligand 1; CI, confidence interval; Sq, squamous cell carcinoma; NA, not available; NSCLC, non-small cell lung cancer; CBDCA, carboplatin; PTX, paclitaxel; PEM, pemetrexed; BEV, bevacizumab.
